# Improving Cognitive Behaviour Therapy for Autistic Individuals: A Delphi Survey with Practitioners

**DOI:** 10.1007/s10942-022-00452-4

**Published:** 2022-04-08

**Authors:** Debbie Spain, Victoria Milner, David Mason, Hannah Iannelli, Chris Attoe, Ruwani Ampegama, Lorcan Kenny, Aleks Saunders, Francesca Happé, Karina Marshall-Tate

**Affiliations:** 1 Social, Genetic and Developmental Psychiatry Centre, Institute of Psychiatry, Psychology & Neuroscience, King’s College London, de Crespigny Park, PO Box 80, London, SE5 8AF England, UK; 2 South London & Maudsley NHS Foundation Trust, London, England, UK; 3 Autistica, London, England, UK

**Keywords:** Autism, Cognitive behaviour therapy (CBT), Practitioners, Training, Clinical supervision

## Abstract

There is emerging evidence of the effectiveness of individual and group cognitive behaviour therapy (CBT) for autistic individuals, in particular to address anxiety, obsessive compulsive disorder and depression. Many CBT studies have incorporated relatively stringent standards, with regards to participant inclusion/exclusion criteria, delivery of manualised approaches and assurance of therapist training and oversight. We know less about what happens in routine CBT practice and, importantly, how service provision can be improved for autistic individuals. The present study recruited 50 CBT practitioners to a three round Delphi survey. The aims were to elicit professionals’ perspectives regarding barriers to the acceptability and effectiveness of CBT for autistic individuals, and to generate consensus, both about ways of enhancing service provision, as well as the autism-relevant training needs of CBT practitioners. Study findings indicated six barriers to accessible and effective CBT for autistic individuals, relating to service provision, practitioner-related factors, client-related factors, CBT-related factors, national guidelines, and systemic considerations. There was participant consensus that changes in five domains (specifically relating to process issues, service provision, practitioners, techniques and therapeutic approach) could improve the CBT care pathway. Consensus was generated about the training needs of CBT practitioners: training about autism, CBT-specific issues, co-occurring conditions and engagement, were deemed fundamental for enhancing practice. Participants also identified autism-relevant issues for clinical supervision. Further sustained research is needed to determine the effects of adapted service provision and improved practitioner knowledge and skills on the outcomes of autistic individuals who have CBT.

## Introduction

Autism spectrum disorder (henceforth referred to as autism), is a lifelong neurodevelopmental condition affecting 1–2% of the population (Roman-Urrestarazu et al., 2021). Main traits of autism include social communication differences, hypo- and hyper-sensory sensitivities, difficulties managing change and uncertainty, circumscribed interests and adherence to routines (APA, 2013). Autism is a spectrum condition. Autistic traits can be subtle or marked, and impact functionally day-to-day to varying degrees. Gender differences are noteworthy, with males diagnosed more commonly than females, at an estimated ratio of 3:1 (Loomes et al., 2017).

The majority of autistic individuals experience at least one co-occurring mental health condition (Lai et al., 2019). Rates of anxiety and affective disorders, eating disorders, psychosis and traumatic stress, for example, are higher in autistic individuals than non-autistic individuals (see Hossain et al., 2020 for an umbrella review). Deliberate and unintentional self-harm, and suicidality, are also common experiences for autistic adults (Cassidy et al., 2020). Poor mental health can compound problems with completing education and training, finding and sustaining employment, developing social connections and, ultimately, attaining independence.

There have been concerted efforts to develop evidence-based psychological therapies for autistic individuals who experience mental health conditions. Individual and group cognitive behaviour therapy (CBT) have been empirically tested most widely. There is emerging evidence of effectiveness of CBT for autistic young people and adults, when compared to treatment as usual, or a wait list or active control (for comprehensive reviews, see Perihan et al., 2020; Spain, 2019; Ung et al., 2015; Weston et al., 2016). Meta-analyses of randomised controlled trial (RCT) data, predominantly describing samples of young autistic people with anxiety, indicate moderate effect sizes for improvement following CBT (Kreslins et al., 2015; Perihan et al., 2020; Ung et al., 2015).

CBT often requires adaptation, so that this is better tailored to the needs and preferences of autistic individuals; for example, to accommodate core autism traits, and impairments commonly associated with autism, such as alexithymia (difficulty identifying own emotions), and difficulties with perspective-taking and emotion regulation (Gaus, 2018; Stark et al., 2021). Adaptations most frequently cited in the literature include: providing more sessions than is standard and slowing down the pace of these; scaffolding emotion recognition and regulation skills; focusing on wider skills development (e.g., social skills interventions, problem-solving strategies, assertiveness); making abstract constructs more explicit; incorporating visual means to share information; using more concrete and didactic methods; emphasising behavioural rather than cognitive interventions and techniques; building special interests into therapy (e.g., within the formulation or when developing a hierarchy of anxiety-provoking situations); and involving parents or caregivers as co-therapists (Kerns et al., 2016; Moree & Davis, 2010; NICE, 2013; Spain & Happé, 2020). Few studies have systematically examined the impact of adaptations on autistic participants’ outcomes after having CBT. However, there is preliminary empirical support for offering extended courses of therapy and having caregiver involvement (Perihan et al., 2020), and augmenting group approaches with individual sessions (Kreslins et al., 2015).

Importantly, many autistic individuals are unable to access timely needs-led assessment and interventions for co-occurring mental health conditions, due to a range of systemic barriers (Maddox et al., 2020a). Recent systematic reviews highlight that these may include service-related factors (e.g., long waiting lists, provision not aligned to the needs of autistic individuals), practitioner-related factors (e.g., lack of knowledge about autism, limited confidence to work with autistic individuals) and client-related factors (e.g., difficulties describing current problems or internal states, sensory sensitivity and overload) (for review, see Adams & Young, 2020; Walsh et al., 2020; see also Maddox et al., 2020a, b).

Limited knowledge, confidence and skills to work with autistic individuals, has been consistently noted by clinical practitioners (for review see Corden et al., 2021). However, a paucity of studies have focused on CBT practitioners’ experiences specifically. As a notable exception, Cooper et al. (2018) examined psychological therapists’ experiences, skills and confidence in working with autistic individuals and adapting their practice. Results indicated a statistically significant relationship between level of training and practitioner confidence, whereby better confidence was associated with higher level of training.

In summary, evidence suggests there can be barriers to autistic individuals accessing appropriately adapted CBT in routine practice. However, there is a lack of systematic research about how the care pathway should be adapted and what would help CBT practitioners to have more knowledge, skills and confidence to offer this. Prior studies (e.g., Spain & Happé, 2020) have recruited practitioners with specific experience and expertise of offering CBT to autistic individuals. It is also important to clarify the perspectives of practitioners who may be working with autistic individuals within mainstream settings, and have limited, moderate or substantial experience of offering autistic individuals CBT. Using an iterative approach, this study aimed to: (1) Determine practitioners’ perceptions of the barriers to acceptability and effectiveness of CBT for autistic individuals; and (2) Develop a consensus statement about ways of enhancing the CBT care pathway for autistic individuals, as well as the autism-related training needs of practitioners.

## Methods

### Study Design and Development

We conducted a Delphi survey, a study design that is useful for gathering perspectives and generating consensus from participants about under-researched topics (Langlands et al., 2008). Delphi surveys are typically conducted over three rounds. Each round builds on the last. Participant responses to Round 1 are synthesised into a Round 2 survey; for example, participants are asked to individually identify essential components of a treatment in Round 1, and their responses are pooled together and refined into a collective list of components (statements) in the second survey. The same participants are then asked in Round 2 to rate the degree to which each component is essential to treatment, so as to generate group consensus. Any statements that fail to reach a consensus score may be re-rated in a third round. Delphi surveys can be resource and time efficient. They are easily completed online, making it possible to recruit from a wide sampling frame. Delphi surveys are also structured in a way that enables participants to have anonymity (i.e., as the pooled, rather than individual, responses are circulated). This may allow participants to be more open as there is less concern about what colleagues or peers may think.

The initial Round 1 survey was developed based on a previous synthesis of the literature relating to good practice guidelines for CBT and seminal texts about CBT for autistic individuals, collated for a similar Delphi survey study (for specific detail, see Spain & Happé, 2020), as well as more recent autism and CBT literature (Maddox et al., 2020a; Perihan et al., 2020; Spain, 2019; Ung et al., 2015) and clinical experience. This also incorporated collaboration with autistic adults and a parent of two autistic children.

In Round 1, participants were asked to: (1) List barriers they considered affect accessibility and effectiveness of the CBT care pathway for autistic individuals, and ways of ameliorating these; (2) Describe any autism-relevant training they had attended; and (3) Highlight gaps in autism-relevant training for CBT practitioners and outline any additional training they thought would be useful for enhancing CBT service provision and practice.

Round 1 responses were synthesised using thematic analysis (Braun & Clark, 2021), in order to develop the Round 2 survey. This comprised 67 statements relating to potential ways of enhancing CBT service provision, an outline of training themes that could address CBT practitioners’ training needs, and considerations associated with CBT clinical supervision. Statements from the Round 2 survey are listed in Tables 2, 3 and 4, in the column marked “Round 2”.

The Round 3 survey consisted of 21 statements: 13 statements that had not gained consensus in the previous round, and eight extra statements that participants had proposed in Round 2. Statements from the Round 3 survey are listed in Tables 2, 3 and 4, in the column marked “Round 3”. Statements were rated on a 5-point scale, as described elsewhere (see Langlands et al., 2008): (1) Essential; (2) Important; (3) Do not know/it depends; (4) Unimportant; and (5) Do not include.

Each survey also included a limited number of demographic questions, designed to contextualise participants’ responses; for example, establishing professional discipline, the work setting, age group of clients seen, and years of experience of working with autistic individuals. Each of the surveys was usability tested by clinicians, an autistic PhD student and members of the team. This resulted in a few modifications, such as amalgamating statements pertaining to overarching points and refining wording of statements for brevity.

### Recruitment

This was an opt in study. We recruited participants using convenience and snowballing sampling, between September and December 2020 (during the global COVID-19 pandemic), principally via existing networks, social media and word of mouth. Only people who had participated in the previous round were contacted regarding the next survey (i.e., the survey was closed to new participants after Round 1).

### Ethical Approvals

Ethical approvals were obtained [REC reference KCL HR - 19/20 - 17744]. All participants gave informed consent.

### Procedure

The survey was designed in Qualtrics, accessible via any internet-enabled device and completed at a time that suited participants. Participants were asked to create a unique ID and passcode, so that they would be able to withdraw their data if they wished to (none opted to do so). Survey completion was estimated as 10–15 min, with each survey taking progressively less time. In Rounds 2 and 3, participants were sent a synthesis of the previous round’s results along with the link to the subsequent round. Participants could opt in to a prize draw with five people winning £25 gift vouchers per round.

### Data Analysis

We summarised demographic information using frequencies, ranges of scores and the means for these. We determined the number of participants who rated statements similarly and evaluated whether these attained group consensus. Parameters for consensus were based on work by Langlands et al. (2008): (1) Statements rated as essential or important by approximately ≥ 80% of participants were considered essential; (2) Statements attaining a consensus rating of essential or important by approximately 60–79% of participants were re-rated once more in Round 3; and (3) Statements not meeting these criteria were not considered integral. As sample sizes differed, the number of participant responses required for consensus varied slightly. Percentages were rounded up to the nearest whole number.

## Results

### Participant Demographics

See Table 1 for an overview of participants’ professional disciplines and the age group of individuals they worked with, across all three surveys. Fifty participants completed the Round 1 survey, 25 completed the Round 2 survey and 11 completed the Round 3 survey.

**Table 1:** Participant demographics

	Round 1 (n = 50)	Round 2 (n = 25)	Round 3 (n = 11)
*Professional discipline*			
Clinical psychologist	19	10	4
Cognitive behavioural therapist	17	9	5
Psychologist or CBT trainee	5	4	-
Psychological wellbeing practitioner	4	1	-
Assistant psychologist	2	-	-
Social worker	1	1	1
Psychiatrist	1	-	-
Psychotherapist	1	-	1
*Population worked with*			
Children and adolescents	9	5	1
Adults	23	9	5
Lifespan	18	11	5

Some additional demographic information was obtained in the Round 1 survey. Approximately 70% of participants provided details about their work setting, which included child and adolescent or adult psychological therapies services, mental health outpatient / community services, independent practice, national specialist services for autistic individuals or for individuals with a learning disability, and education and forensic services. There was variation in the standard number of sessions offered to clients presenting for CBT, depending on the service they worked in: several services offered between 6 and 12 sessions, with fewer services offering 12–24 sessions. Forty-eight percent of participants (n = 24) worked in services that automatically capped the number of sessions offered to individuals.

Participants had used CBT as part of their clinical and/or academic role for between one and 22 years (mean number of years of CBT experience 8.4 years). The proportion of people on participants’ current caseloads who were autistic ranged from 0 to 100% (mean % of individuals on caseload diagnosed with autism 28%); approximately 60% of practitioners worked with autistic individuals less than 25% of the time, and 20% of practitioners worked with autistic individuals for between 26 and 50% of the time. Seventy four percent of participants (n = 29) sometimes worked with individuals who were suspected to be autistic but not formally diagnosed. Sixty percent of participants also worked with individuals with a learning disability. When asked, 74% of participants (n = 37) reported that individuals they worked with were not generally concurrently being seen by another health service, excluding the GP.

### Barriers to Acceptability and Effectiveness of CBT

In the Round 1 survey, participants were asked to identify any barriers to the acceptability and effectiveness of CBT for autistic individuals. Data were analysed thematically (Braun & Clark, 2021). Two members of the research team independently reviewed participants’ responses and assigned codes and then categories to these. They then discussed overarching names for themes. Analysis indicated that barriers reported could be categorised into six main themes. These are ranked from the most to least frequently reported barriers:

1. Factors relating to service provision (e.g., long waiting times, lack of resources, autistic individuals deemed ineligible or too complex for services, the referral and triage system could seem inappropriate for individuals with social communication needs, insufficient number and duration of sessions, limited reasonable adjustments offered);
2. Practitioner-related factors (e.g., lack of understanding of autism, diagnostic overshadowing, lack of knowledge about how to adapt interventions and techniques, lack of flexibility in approach, insufficient training, limited confidence to work with autistic individuals, perceptions that CBT may not be a suitable therapy for autistic individuals);
3. Client-related factors (e.g., multimorbidity, difficulties with articulating thoughts and feelings, theory of mind impairments, sensory sensitivities, cognitive rigidity, difficulties tolerating change and generalising skills, adverse past experiences of therapy or service provision);
4. CBT-related factors (e.g., standard treatment protocols may not apply, difficulties developing a therapeutic alliance, short-term tangible goals seeming difficult for autistic individuals to identify or articulate, metaphors and analogies seeming inaccessible for autistic individuals, conceptual questions about whether coping strategies are adaptive responses or safety-seeking behaviours);
5. Factors relating to national guidelines (e.g., the National Institute for Health and Care Excellence (NICE) guidelines not fully encapsulating what autistic individuals may benefit from); and
6. Systemic considerations (e.g., family not involved, poor links between health services, stigma).

### Factors that Could Deter Practitioners from Offering CBT to Autistic Individuals

In the Round 2 survey, and in addition to the statements outlined below, we asked participants about factors that might deter them from offering CBT to autistic individuals. Responses were clustered into five themes, as follows:

1. Compatibility of individuals’ needs and the service remit (e.g., whether a service that provides short-term CBT could offer what was clinically needed), and metrics used to measure change (e.g., whether standardised outcome measures would reliably demonstrate improvement and recovery if it was not possible to identify a tangible end to intervention);
2. Appropriateness of the intervention (e.g., why the referral had been made and by whom, whether CBT seemed indicated as compared to another modality such as family-focused interventions);
3. Practitioner-related factors (e.g., if unable to provide CBT with regularity);
4. Client-related factors (e.g., co-occurring intellectual disability, poor functioning at the time of the referral, if individuals declined to engage); and
5. Systemic factors (e.g., poor family support, evidence of ongoing abuse, adverse social circumstances).

### Enhancing CBT Service Provision

Participants made numerous suggestions for ways the CBT care pathway could be adapted to better meet the needs and preferences of autistic individuals, thereby potentially making it more accessible and interventions more effective. Suggestions were encapsulated into 34 statements that were included in the Round 2 and/or Round 3 surveys (see Table 2). Statements pertained to five areas, also ranked according to how frequently themes were reported by participants:

1. Process issues (e.g., use of visual prompts, frequent repetition, adapting worksheets);
2. Service provision (e.g., easier and more rapid access to an autism diagnostic assessment, joint working and better links between services, an adapted triage system);
3. Practitioners (e.g., increased practitioner knowledge and understanding of autism, having time to conduct or read relevant research);
4. Techniques (e.g., using a formulation-based rather than protocol-derived approach, offering psychoeducation, teaching distancing techniques); and
5. Therapeutic approach (e.g., capacity for working with families or the wider system, access to third wave approaches).

**Table 2:** Enhancing CBT provision for autistic individuals

Enhancing the CBT care pathway: How important are the following for enhancing the CBT care pathway for autistic people?				
	Round 2	Round 3	Percentage agreement	Domain
Increased practitioner knowledge and understanding of autism	√		100	P
Easier more rapid access to an autism diagnostic assessment	√		92	SP
Joint working and better links between services	√		76	SP
Autism pathways within mainstream mental health services	√	√	100	SP
Flexibility in the service model offered (e.g., flexible DNA policy, personalised pathway, range of therapies offered, mode of delivery)	√		96	SP
Flexibility in Key Performance Indicators measured	√		84	SP
An adapted triage system (e.g., less emphasis on brief phone assessments and screening forms)	√		80	SP
A longer assessment phase	√	√	82	SP
Clarifying clients’ thoughts, beliefs and expectations about CBT	√		88	Pr
Offering group interventions	√	√	73	TA
Capacity for working with families or the wider system	√		80	TA
Flexibility in the number, duration and frequency of sessions	√		100	SP
Accommodation of sensory preferences	√		100	Pr
Using a protocol-driven approach	√	√	54	T
Using a formulation-based approach	√		88	T
Offering psychoeducation (e.g., about emotions)	√		100	T
Sessions focusing on what an autism diagnosis means to the client	√	√	82	T
Use of visual prompts (e.g., charts, diagrams)	√	√	82	Pr
Frequent repetition (e.g. of concepts, tasks, techniques)	√	√	91	Pr
Avoiding use of metaphors	√		52	Pr
Providing written information to take home	√		84	Pr
Adapting worksheets (e.g., from the standard psychoeducational or homework forms routinely given out during sessions)	√		84	Pr
Using a predominantly behavioural approach	√		28	TA
Using a predominantly cognitive approach	√		84	TA
Setting up more opportunities for generalisation of skills, than might be usual	√		92	Pr
Doing things outside the therapy room (e.g., experiments, exposure)	√		92	Pr
Explicitly discussing how to address obstacles to progress and therapeutic ruptures	√		92	Pr
Using idiosyncratic rating scales (e.g., those that are colourful, visual)	√	√	76	Pr
Incorporating ‘special interests’ within therapy	√	√	64	Pr
Having time to conduct, or read, relevant research	√		88	P
Providing psychoeducation about the freeze situation and how this relates to social situations		√	82	T
Access to third wave approaches		√	64	TA
Teaching distancing techniques		√	36	T
Embedding creativity in CBT methods		√	82	Pr

*SD *service provision; *P* practitioners; *TA* therapeutic approach; *Pr* process; *T* techniques

Overall, 25 out of 34 statements were considered essential or important for reducing barriers and enhancing CBT provision for autistic individuals.

### Autism-Relevant Training Previously Attended

Seventy two percent of participants (n = 36) had attended at least one autism-specific training event during initial clinical training or since qualifying, primarily seminars, workshops and study days. Common descriptions of the contents of training included autism awareness, autism diagnostic assessment, sensory processing, mental health in autism and CBT for autistic individuals. The latter training had been attended by 28% (n = 14) of participants. Training courses attended had been delivered for between two hours and five days, depending on the focus, and whether this comprised formal teaching (e.g., during core training or a workshop), or a conference event.

### Training Gaps and Needs

Participants considered that improving the amount and content of autism-relevant training for CBT practitioners would be beneficial for working with autistic individuals. A modular approach to training (e.g., with mix and match sessions), delivered via different mediums (e.g., e-learning, in person sessions, experiential groups), was favoured by many.

Participants identified 20 training topics that seemed relevant for CBT practitioners (see Table 3). These related to four areas, ranked in order of those themes mentioned most to least frequently by participants:

1. Autism (e.g., an overview of core autism symptomatology, cognitive theories of autism, assessment and diagnosis of autism);
2. CBT-specific issues (e.g., developing an idiosyncratic formulation that takes into account core and co-occurring symptoms, adapting standard treatment protocols so these are better tailored for autistic people, adapting the process of CBT);
3. Co-occurring conditions (e.g., neuropsychological traits associated with autism, assessment, diagnosis and treatment of co-occurring mental health symptoms, understanding and working with attachment-based difficulties); and
4. Engagement (e.g., enhancing communication and engagement, understanding and addressing barriers to engagement).

**Table 3:** Integral components of autism-relevant training for CBT practitioners

Training: How important are the following training topics for practitioners using CBT with autistic people?				
	Round 2	Round 3	Percentage agreement	Domain
An overview of core autism symptomatology, including in people with differing presentations (e.g., males and females)	√		96	A
Cognitive theories of autism	√		92	A
Assessment and diagnosis of autism	√		80	A
The experience and impact of autism	√		92	A
Neuropsychological traits associated with autism (e.g., cognitive style, difficulties with perspective taking), and ways to accommodate these	√		100	C
Formulating and addressing alexithymia	√		84	C
Formulating and addressing an intolerance of uncertainty and difficulty with change/transition	√		96	A
Assessing and accommodating sensory preferences, sensitivities and aversions	√		96	A
Understanding and addressing behaviour deemed ‘challenging’	√		92	C
The concept of camouflaging / masking	√		96	A
Enhancing communication and engagement	√		100	E
Assessment, diagnosis and treatment of comorbid mental health symptoms	√		96	C
Developing an idiosyncratic formulation that takes into account core and comorbid symptoms	√		96	CBT
Adapting standard treatment protocols so these are better tailored for autistic people	√		92	CBT
Adapting CBT interventions to enhance acceptability and effectiveness	√		96	CBT
Understanding and addressing barriers to engagement	√		100	E
Understanding and working with attachment-based difficulties	√	√	82	C
Designing and delivering interventions for autism traits (e.g., post-diagnostic support, social skills interventions)	√		80	A
Assessing suitability for therapy and developing preparedness for therapy		√	100	CBT
Adapting the process of CBT (e.g., how to communicate effectively, set up sessions in an autism-friendly way)		√	100	CBT

*A *autism; *C *co-occurring conditions; *CBT *CBT-specific issues; *E *engagement

All 20 topics were considered essential or important for enhancing CBT practitioners’ practice.

### Considerations for Supervision

A number of participants identified autism-relevant considerations for CBT clinical supervision, that were refined into 21 statements (see Table 4). Seventeen statements were deemed essential or important aspects of clinical supervision when working with autistic individuals. These were clustered into four areas, and are listed according to the themes reported most to least frequently:

1. Suggestions for focal discussion points during supervision (e.g., about thoughts, assumptions or beliefs about autism, formulation of therapy-interfering factors, consideration of idiosyncratic risk factors);
2. Considerations for clinical supervisors (e.g., knowledge of core autism symptoms, experience of using CBT with autistic individuals, confidence in working with autistic individuals); and
3. Considerations for clinical supervisees (e.g., knowledge of core autism symptoms, knowledge of characteristics associated with autism).
4. Oversight issues (e.g., use of the Cognitive Therapy Scale – Revised);

**Table 4:** Integral components of clinical supervision for CBT practitioners

Supervision: How important are the following facets for clinical supervision?				
	Round 2	Round 3	Percentage agreement	Domain
Supervisee knowledge of core autism symptoms	√		92	Se
Supervisee knowledge of characteristics associated with autism (e.g., impairments in neuropsychological functioning, differential diagnoses)	√		96	Se
Supervisee knowledge of autism presentations in males and females	√		96	Se
Supervisee good knowledge of mental health needs associated with autism		√	91	Se
Supervisee training in autism	√		88	Se
Supervisor knowledge of core autism symptoms	√		100	So
Supervisor knowledge of characteristics associated with autism (e.g., impairments in neuropsychological functioning, differential diagnoses)	√		100	So
Supervisor knowledge of autism presentation in males and females	√		100	So
Supervisor experience of using CBT with autistic people	√		92	So
Supervisor confidence in working with autistic people	√		96	So
Supervisor good knowledge of mental health needs associated with autism		√	100	So
Discussing cases more frequently than when working with autistic people	√	√	54	O
Extended supervision sessions to discuss clinical work	√		36	O
Use of the Cognitive Therapy Scale – Revised	√		20	O
Opportunities to discuss thoughts, assumptions or beliefs about autism	√		92	O
Discussion about autism-relevant adaptations to the structure and process of therapy	√		100	D
Discussion about autism-relevant adaptations to CBT interventions and techniques	√		96	D
Formulation of therapy-interfering factors	√		100	D
Discussion about non-standardised outcome measures to use	√	√	100	D
Consideration of systemic influences (e.g., contributing to the formulation, engagement, outcomes, family distress)	√		88	D
Consideration of idiosyncratic risk factors (e.g., to self, others, from others)	√	√	100	D

*Se *supervisee; *So *supervisor; *D *discussion points; *O *oversight

## Discussion

Emerging evidence indicates that autistic individuals can benefit from CBT. The present study focused on eliciting practitioners’ perspectives about barriers to the accessibility and effectiveness of CBT, and generating consensus about ways of enhancing the CBT care pathway as well as practitioner knowledge, understanding and skill, so that service provision is more honed to the needs and preferences of autistic individuals.

Findings from the present study indicate that the most prominent barriers to CBT concern service provision and practitioner-related factors, and to a somewhat lesser extent, client-related factors and CBT-related factors. This implies that *how* CBT is offered, *the context within which this takes place* and *by whom* (i.e., the amount of autism-relevant knowledge, skills and confidence practitioners have), can influence access to and, potentially, effectiveness of, therapy for autistic individuals.

This mirrors findings reported elsewhere. In a review of 12 studies describing barriers and facilitators to psychological therapy for autistic individuals, Adams and Young (2020), for example, identified similar problems, such as services seeming inaccessible, autistic individuals falling between ‘gaps’ in provision and not being ‘listened to’, poor practitioner knowledge and skills, and a lack of tailoring of interventions and techniques. Barriers have also been described in the context of access to physical healthcare for autistic individuals, and include communication disconnect between practitioners and autistic individuals, the impact of sensory sensitivities, and practical issues associated with executive functioning (Mason et al., 2019). Additionally, findings are supported by a very recent survey study of 537 autistic adults that examined accessibility of physical and mental healthcare provision, and outlined similar barriers to care (Brice et al., 2021).

Importantly, consensus among Delphi survey participants indicated that the CBT pathway can be enhanced in several ways. Changes to the process of CBT, service provision and practitioner knowledge, skills and confidence were mentioned most frequently by participants. Again, this reinforces the idea that the context within which CBT is provided and the process by which therapy is offered, are of pivotal importance. These findings are comparable with previous Delphi surveys focusing on CBT for autistic individuals (Spain & Happé, 2020) and facilitators for psychological therapy for autistic individuals more widely (Adams & Young, 2020).

Taken together, study findings indicate that practitioner knowledge and skill is important for accessible and effective CBT. Indeed, perceived self-efficacy has been found to influence volition to offer CBT to autistic individuals, whereby poorer knowledge and confidence about autism decreased the chances of offering CBT, even where this was potentially clinically indicated (Maddox et al., 2020a). Approximately 70% of participants in the present study had previously attended autism-relevant training, although the content, duration and level appeared to vary substantially. In a study focusing on practitioner knowledge, skill and confidence to use CBT with autistic individuals, Cooper et al. (2018) reported that 36% (n = 18) of their sample of therapy practitioners had previously attended autism-relevant training. They also reported a statistically significant relationship between level of training and practitioner confidence to work with this clinical group.

Consensus amongst participants here suggests that training about general or overarching topics associated with autism and co-occurring difficulties/conditions was deemed important, in addition to training about how to design and deliver adapted CBT. The Oliver McGowan Mandatory Training in Learning Disability and Autism (HEE, 2020) is a recent England-wide initiative that aims to ensure public facing workers have training in autism and learning disability. Currently in set up, this will comprise tiered training that ranges from general autism awareness to more specialist skills (e.g., adapting CBT). Findings from the present study may help to inform training themes.

Finally, we found that there are autism-relevant issues associated with clinical supervision, for both supervisees and supervisors. Clinical supervision is a cornerstone of clinical work. Specific competencies for CBT supervision when working with non-autistic individuals have been outlined (e.g., Roth & Pilling, 2008), with a view to ensuring practitioners demonstrate fidelity to central CBT tenets, make formulation-guided choices about interventions and techniques and show awareness of non-specific factors associated with CBT practice, such as the importance of the therapeutic alliance. One of the conceptual and practical complexities associated with offering CBT to autistic individuals is that there can either be deviations from fundamental principles or use of different procedures (Gaus, 2018; Spain, 2019); for example, using a more didactic approach, or emphasising behavioural interventions even when cognitive work is more typically indicated. To our knowledge, no studies have systematically investigated whether content, process or perceived quality and salience of CBT clinical supervision influence outcomes for autistic individuals. Yet the views gathered here suggest that knowledge of autism and associated conditions, and their impact on the therapy context and process, is needed to ensure that focal points for supervision and oversight are autism-relevant.

The overall implication from the study findings is that barriers and facilitators to CBT, and indeed health services more generally for autistic individuals, may be best viewed systemically and multidimensionally. Understanding these in general terms means that service provision can be improved globally. Yet there may also be a need to identify the specific barriers and facilitators for each autistic individual; for example, taking sensory sensitivities into account may be necessary for some, whereas accommodating information processing difficulties/differences may be relevant for others.

CBT practitioners should take the time at the outset of a course of therapy to find out more about what clients prefer or find difficult to tolerate, so that, collaboratively, they can identify strategies. Alternatively, some autistic individuals find it useful to develop an autism (communication) passport, that outlines needs, preferences and coping strategies. Health passports are used relatively commonly within inpatient (ward) settings, but seemingly less so within community-based services. However, this is one way of establishing how support can best be tailored for autistic individuals. With consent, this can be shared across health contexts, so that individuals do not need to keep retelling this information.

We acknowledge several study limitations. As is common with online surveys circulated via methods including social media, we could not estimate the reach of the survey, and therefore the proportion of people who saw the study advertised versus the number of people who took part. Selection bias is possible; we did not ask about motivations for participating. The sample size was small, and this may therefore affect generalisability of findings. However, recruitment took place during the first year of the COVID-19 pandemic (during the time of pandemic-related restrictions and more burden on health services), and therefore, the sample size seemed pragmatic. Participants’ knowledge of autism, the amount and years of experience of working with autistic individuals with or without an intellectual disability, and qualifications and proficiency as CBT practitioners were not measured. These factors may have influenced suggestions identified or endorsed during consensus building. We did not provide descriptions of specific types of autism training events participants may have attended (e.g., distinctions between a workshop and seminar), and so it is possible that participants may have referred to training events similarly or differently. Additionally, the dataset did not allow for sub-group analysis, such as examining whether frequency of using CBT versus other psychological therapies differed according to how often participants worked with autistic individuals. Finally, as is very common in Delphi surveys, attrition between rounds was an issue.

In summary, and reflecting findings from the literature, data reported here indicate that changes to service design and the process and content of CBT could, in the views of practitioners, mitigate barriers autistic individuals encounter when accessing therapy. Importantly, participants considered that enhancing knowledge of autism and co-occurring conditions, as well as practitioners’ skills and confidence, may be pertinent for improving the CBT care pathway for autistic individuals. More research is needed to establish the impact of autism-relevant adaptations and improved practitioner knowledge on accessibility and effectiveness of CBT.

## Data Availability

The raw data for the study are not available as sharing of this was not requested as part of the ethical approvals process. More information about the study, methods and data can be obtained from the corresponding author.
